# Vascular complications related to Le Fort I osteotomy: a scoping review

**DOI:** 10.4317/medoral.27277

**Published:** 2025-08-16

**Authors:** Luiza Clertiani Alves, Joana Maria Alves, Ariana Maria Soares, Delane Gondim

**Affiliations:** 1 Postgraduate Program in Dentistry, Faculty of Pharmacy, dentistry and Nursing, Federal University of Ceará; 2 Postgraduate Program in Morphofunctional Sciences, Department of Morphology, Faculty of Medicine, Federal University of Ceara

## Abstract

**Background:**

Le Fort I (LFI) osteotomy represents a secure procedure employed for correcting facial deformities and may be coupled with other facial osteotomies. However, notwithstanding professional proficiency, this technique is not exempt from issues or morbidities. Therefore, the objective of this study was to relate the types of vascular complications associated with LFI.

**Material and Methods:**

A scoping review was conducted with published articles up to April 2024 sourced from PubMed, LILACS, EMBASE, Scopus, Web of Science, Cochrane Library and Google Scholar. The analyzed data comprised: i) comprehensive details pertaining to each vascular lesion associated with LFI; and ii) pertinent anatomical characteristics along with their implications.

**Results:**

A total of 2,415 papers were identified. After removing duplicates and applying the inclusion and exclusion criteria, 33 studies were selected. All included patients had dentofacial deformities. The observed vascular lesions or alterations included: pseudoaneurysm, arteriovenous fistula, section or laceration, maxillary avascular necrosis, internal carotid artery dissection, middle cerebral artery ischemia, and cervicofacial hematoma. Notably, pseudoaneurysm emerged as the most prevalent complication. The surgical features associated with these complications included the incision of the maxillary bone, extending upwards from the pterygomaxillary junction to the pyriform aperture and involving the lateral walls of the nasal cavity. Additionally, disruption of the nasal septum was noted. The internal maxillary artery and its terminal branches are the most exposed to the surgical trauma.

**Conclusions:**

Understanding the potential complications, their clinical presentation, diagnostic methodologies, and management options is of paramount importance. Moreover, a multidisciplinary approach is frequently required to effectively address these complexities.

** Key words:**Le Fort I osteotomy, maxillary surgery, vascular complication, dentofacial deformities, surgical technique, surgical procedure.

## Introduction

The Le Fort I (LFI) fracture pattern, first described by Rene LeFort in 1901 and initially indicated for accessing skull base tumors, was first used in the context of facial orthopedics by Wasmund in 1921. The current LFI osteotomy standard consists of cutting the maxillary bone from the lateral wall of the nasal cavity, breaking the nasal bone septum, to the pterygomaxillary junction (PTJ), allowing the maxilla to move in all three planes, as well as correcting hypoplasia of the middle third of the face, vertical excess of the maxilla and dentofacial asymmetry (1,2).

The LFI osteotomy is considered a safe, efficient and predicTable procedure, and most of the complications are reversible and without permanent sequelae. However, it is not free of serious complications with risk of death (1,3). In this case, vascular complications have been reported during and after this type of osteotomy, appearing as excessive bleeding, compressive effects and blood flow changes (4).

Due to the severity of some of these complications and ethical issues, research and reports may be underestimated and there are no studies showing the compilation of information. For this, the data about the types of possible vascular complications involved, and their prevalence and management must be assessed, as well as the most affected blood vessels and the most likely causal factors. In addition, maxillofacial surgeons must recognize the potential complications associated with the procedure, and the initial symptomatology and course of the lesion, aiming at the early management of these alterations and thus avoiding severe and irreversible outcomes.

## Material and Methods

-Protocol and registration

This scoping review was based on the five-step methodology proposed by Arksey and O’Malley and followed the PRISMA Extension for Scoping Reviews (PRISMA-ScR) (5,6).

- Research question

The starting question was: "What types of vascular complications are associated with LFI osteotomy?" and it was registered under https://osf.io/s5mu6.

- Identification of relevant studies

The literature search was performed in PubMed, LILACS, EMBASE, Scopus, Web of Science, Cochrane Library, and Google Scholar databases for articles published up to April 2024, using medical term descriptors (MeSH) matched by the Boolean operators "OR" and "AND": “Osteotomy, Le Fort” OR “Le Fort Osteotomy” OR “LeFort Osteotomy” OR “Le Fort I Osteotomy” OR “Maxillary expansion” OR “Rapid maxillary expansion” OR “Maxillary surgery” OR “Maxillary osteotomy” OR “Maxillary advancement” AND “ischemia” OR “osteonecrosis” OR “Bone necrosis” OR “Bone aseptic necrosis” OR “Bone avascular necrosis” OR “Carotid-Cavernous Sinus Fistula” OR “Carotid Artery Diseases” OR “Cavernous Sinus Fistula” OR “Epistaxis” OR “Nosebleed” OR “Nasal bleeding” OR “Nose bleeding” OR “Hemorrhage” OR “Bleeding” OR “Aneurysm, False” OR “Vascular injury” OR “False aneurysm” OR “Vascular complication” OR "Maxillary Artery" OR "Ophthalmic Artery" OR "Arteries" OR "Veins".

- Study selection

The standard descriptors used in the search strategy were selected to identify descriptive studies of complications associated with LFI osteotomy. The elements of the study question included: Participants (P) = patients with dentofacial deformity or skull base lesions who underwent LFI osteotomy; Exposure (E) = vascular complications related to LFI; and Outcomes (O) = prevalence of vessel injuries, clinical and surgical management, sequelae, and prevention methods.

Inclusion criteria: Case reports and case series involving vascular complications in the middle third of the face following LFI osteotomy, with or without other procedures on the gnathic bones. The selected publication period was the last 21 years up to April 2024, with no language restrictions.

Exclusion criteria: Studies unrelated to the subject; unavailability of the full text; and patients with blood dyscrasias or those using medication that directly affects the blood coagulation system.

The selection process was conducted in two phases: Phase 1 - two researchers (LCVA and JMSA) independently reviewed the titles and abstracts of all identified references, applying the inclusion criteria in a blinded process; Phase 2 - the same reviewers independently applied the exclusion criteria to the remaining studies based on full-text reading, also blinded. Inter-rater reliability during the study selection was determined by the Cohen kappa test, with a satisfactory value of 0.85. Any disagreements at any stage were resolved through discussion with a third reviewer (DVG). The final decision was always based on the full text of the published studies.

- Data collection

The full texts were carefully evaluated. Data from the eligibility forms were stored in Tables using an independent method by LCVA, JMSA, and AMSS. The validation process was conducted by a fourth reviewer (DVG).

- Compilation, summarization, and reporting of results

After data extraction, the findings were presented using a narrative report divided into two main sections: individualized information about the course of vascular injuries associated with LFI osteotomy as described in each report; and the relevance of surgical anatomy and its implications. The following data related to the characteristics and course of the injuries were extracted and detailed in a Table (Table 1): authors and year of publication, type of study, affected blood vessels, signs and symptoms, time between the procedure and onset of symptoms, type of vascular injury, probable cause, and management.

## Results

**Figure 1:**
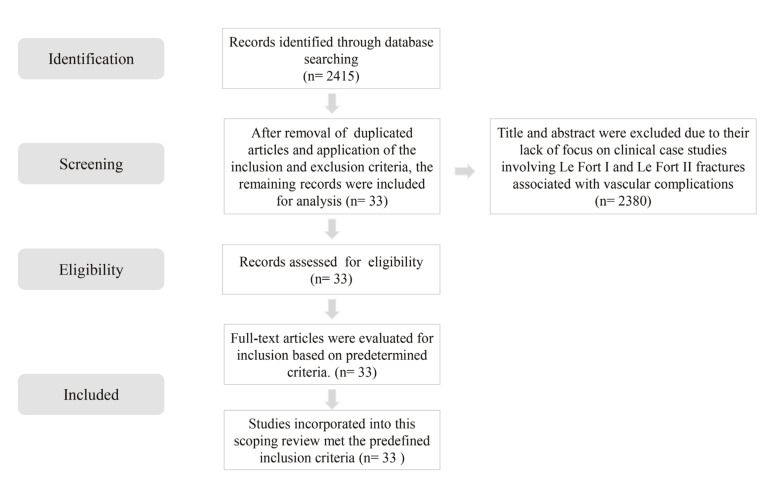
Flowchart of the electronic search.

The initial search retrieved 2,415 articles. After removing duplicates and applying the inclusion and exclusion criteria, a total of 33 articles were selected for analysis (Fig. 1).

The final sample included 41 patients who underwent LFI osteotomy. The female-to-male ratio was 18:22, and the mean age was 26 years, ranging from 14 to 52 years. Most patients had no significant past medical history.

All patients presented with dentofacial deformities requiring correction of the midfacial third. Reported conditions included Angle Class II or III malocclusion, maxillary atresia, facial asymmetry, vertical maxillary excess, and hypoplasia secondary to cleft lip and palate. Maxillary movements were performed to address these deformities, including segmentation, expansion, intrusion, extrusion, advancement, and setback.

The vascular complications reported in the selected articles were as follows: pseudoaneurysm (3,4,7,8,9-19); arteriovenous fistula (20-25) laceration (26,27); partial vascular necrosis (28-32); and total vascular necrosis (33). Additionally, one case of cervicofacial hematoma (34) and one case of internal carotid artery dissection (35) were reported.

The vessels most frequently associated with these complications were the sphenopalatine artery (4,7,10,16,18,27,28), followed by the internal carotid artery (8,17,20,21,35).

**Figure 2:**
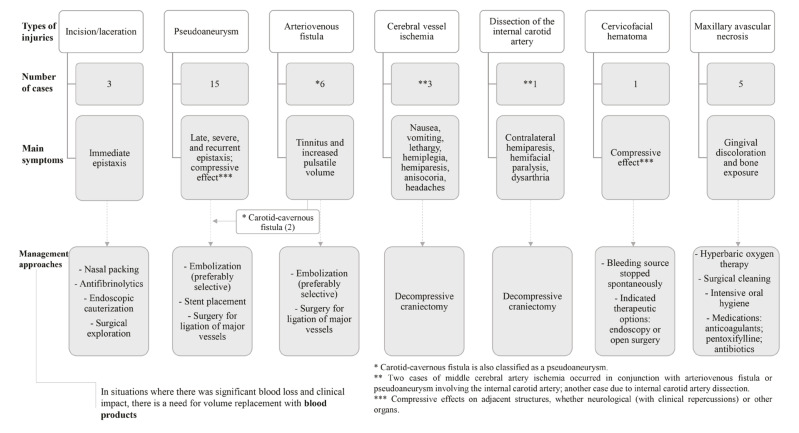
Main vascular injuries associated with Le Fort I osteotomy procedure and their management.

**Figure 3:**
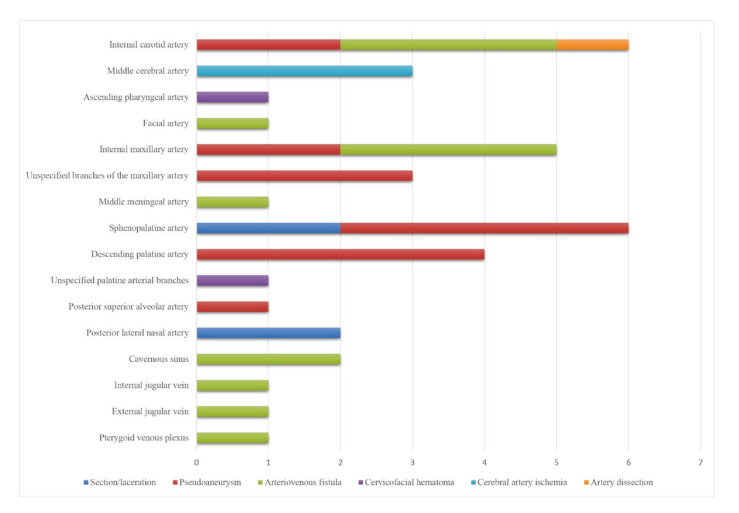
Primary blood vessels affected during the Le Fort I osteotomy procedure.

Detailed information regarding the characteristics and clinical course of these injuries is presented in Table 1, and a graphical summary was generated for quantitative visualization (Fig. 2, Fig. 3).

## Discussion

Deformities of the maxillofacial skeleton are usually corrected through orthognathic surgery, involving the mobilization of the gnathic bones. LFI osteotomy is is a widely performed and generally safe procedure for correcting midfacial discrepancies and may be combined with other surgical techniques. Despite the expertise of the surgical team, the procedure is not without risks. Although complications are rare and, in most cases, reversible, they must be carefully considered (4,7,33).

Complications from orthognathic surgery may present in the short or long term—occurring hours, days, or even weeks postoperatively—depending on the nature of the injury. The most severe complications involve vascular structures and may be life-threatening, regardless of whether they are of arterial or venous origin. In our review, pseudoaneurysm was the most frequently reported vascular complication (3,4,7-19). We believe this frequency in the literature reflects both their clinical relevance and rarity, which prompts publication for educational purposes and to discuss diagnostic and therapeutic strategies.

Epistaxis may occur immediately or at a delayed stage, manifesting as isolated or recurrent episodes, with bleeding ranging from mild to severe (3-5, 7,9-11,13-16,18,26-28,36). Recognizing these patterns helps guide diagnostic suspicion. Direct vascular injuries, such as lacerations, typically cause immediate bleeding, while pseudoaneurysms often develop later, days to months postoperatively, and are frequently associated with recurrent nasal bleeding. Pseudoaneurysms of small branches in deep facial regions, such as terminal branches of the maxillary artery, are rare. Moreover, partial injuries involving only one or two layers of the vessel wall (excluding simultaneous involvement of the intima, media, and adventitia) are also uncommon (7,34,37).

Lesions such as pseudoaneurysms, arteriovenous fistulas, and hematomas can be clinically significant and costly to manage. Even when located in deep anatomical planes and not externally visible, they may compress surrounding structures. Depending on their location, this compression can result in serious symptoms including cranial nerve deficits, ocular disturbances, airway obstruction, central nervous system involvement, or altered cerebral perfusion, leading to signs such as lethargy, dysarthria, hemiparesis, facial paralysis, nausea, and headache (8,9,15,20,21,34,35).

One particularly relevant complication is cervicofacial hematoma, as described by Bertossi *et al*. (2012) (34). It was observed on the first postoperative day after a Le Fort I osteotomy and led to upper and lower airway compression, requiring urgent medical intervention. This highlights the critical importance of continuous postoperative monitoring for the early detection and management of potential

Among diagnostic tools, arteriography was the most frequently employed and effective for identifying pseudoaneurysms and arteriovenous fistulas. When clinical signs suggest vascular lesions, early arteriographic evaluation is recommended due to the unpredicTable course and rupture risk of such injuries. Selective arterial embolization is considered the first-line treatment, as it is minimally invasive, effective, and preserves the vascular architecture more effectively than surgical interventions like vessel ligation (7,10,11,37).

Arteriovenous fistula was the second most frequently reported vascular complication in our review (20-25). Its potentially progressive nature and risk of hemodynamic instability necessitate early detection. Most reported cases responded well to endovascular embolization.

The LFI osteotomy involves an ascending bone cut from the PTJ to the pyriform aperture, along the lateral nasal wall, and includes fracturing the nasal septum. Special attention must be paid to avoid injuring adjacent soft tissues, especially the small and large blood vessels in the posterior nasal cavity and pterygopalatine fossa—regions approximately 2.5 cm from the PTJ (37). These areas are difficult to access and highly vascularized by the internal maxillary artery and its branches, making them particularly vulnerable to surgical trauma (12).

In several cases, the specific arterial branch involved in a complication could not be identified, as many studies did not clearly report which vessel was affected. Nonetheless, among identifiable cases, the sphenopalatine artery was the most commonly involved branch of the maxillary artery (4,7,10,16,18,27,28).

It is important to note that improper execution of the PTJ separation and subsequent down-fracture may generate excessive force on the skull base, potentially resulting in fractures and damage to adjacent structures. Inappropriate angulation of osteotomes, incomplete osteotomy cuts, and improper activation of expansion devices are reported contributors to such complications (6,8,10,13,14,20,22).

After down-fracture, the maxilla's blood supply mainly depends on the ascending pharyngeal, ascending palatine, descending palatine, and small mucosal terminal arteries. There is ongoing debate about whether sacrificing the descending palatine arteries is justified to prevent hemorrhage, especially in complex cases like clefts or syndromic patients. However, refined surgical techniques can often prevent complications while preserving vascular integrity (25,33).

Avascular necrosis of the maxilla (24,29-31,33) is an important complication associated with LFI osteotomy. In most reported cases, it led to partial bone loss, including the loss of teeth and alveolar bone segments, and occasionally resulted in oronasal fistulas. In more severe instances, total maxillary necrosis occurred, causing devastating functional and aesthetics consequences. To reduce the risk of this outcome, it is essential to ensure adequate vascular supply through meticulous surgical planning and techniques such as hypotensive anesthesia.

In conclusion, LFI osteotomy requires precise surgical technique and a thorough understanding of the anatomical landscape, particularly the vascular structures in high-risk regions such as the pterygoid and posterior nasal fossae. Early recognition and diagnosis of vascular complications, along with timely and appropriate interventions coordinated through multidisciplinary collaboration, are essential to minimizing morbidity and optimizing patient outcomes.

## Figures and Tables

**Table 1:** Included studies.

Study (Year of Publication	Type of study	Onset/ Signs and Symptoms	Affected Blood Vessel	Type of Injury	Probable Cause	Management of Vascular Injury
Avelar et al.,2010 (8)	Case Report	63 POD; epistaxis, pain, and edema in the hemiface	Sphenopalatine artery	Pseudoaneurysm	NR	NP + embolization
Bertossi et al.,2012 (34)	Case Report	1 POD; Dyspnea and neck swelling	Palatine arterial branches and ascending pharyngeal artery	Cervicofacial haematoma (laceration)	NR	Amoxicilin+ methylprednisolone
Bouter et al., 2021 (23)	Case Report	IPP; Tinnitus and pulsation, pulsatile mass in the mandibular angle	External jugular vein and branches of the facial artery and internal maxillary artery	Arteriovenous fistula	Iatrogenic	embolization
Bykowski et al., 2018 (13)	Case Report	14 POD; epistaxis	Maxillary artery branches	Pseudoaneurysm	Force for Down-fracture; PTJ separation; bone spicules	NP + volume replacement (2PRBC) + embolization
Carneiro et al., 2013 (20)	Case Report	1 POD; Ptosis (eyelid drooping), blurred vision, ophthalmoplegia, ocular pain (involvement cranial nerves III; IV; V1e VI)	Internal carotid artery and cavernous sinus	Arteriovenous fistula (cavernous carotid fistula)	Basilar skull fracture (use of curved chisel in PTJ or activation of the expansion device)	embolization
Chepla et al., 2010 (3)	Case Report	7 POD; epistaxis	Maxillary artery branches	Pseudoaneurysm	NR	Volume replacement in 2 stages (2PRBC/3PRBC) + embolization
Dermarkarian et al., 2019 (21)	Case Report	1 month; reduced visual acuity; pulsatile tinnitus; binocular diplopia; ocular proptosis; partial ophthalmoplegia	Internal carotid artery and cavernous sinus	Arteriovenous fistula (cavernous carotid fistula)	NR	embolization
Ettinger et al., 2020 (33)	Case Report	2 POD; Darkening of gingival tissues in the maxilla, lip pallor	Unclear	Total avascular necrosis in the maxilla	NR	Hyperbaric oxygen therapy; resection of necrotic tissues; reconstruction with microvascularized graft and implants
Fernández-Prieto et al., 2005 (14)	Case Report	IPP; severe epistaxis (recurring at 21 and 7 days)	Descending palatine artery	Pseudoaneurysm	Moment of PTJ separation and force in Down-fracture	NP + Volume replacement + embolization
Goffinet et al., 2010 (22)	Case Report	1 POD; tinnitus	Internal maxillary artery and venous malformation	Arteriovenous fistula	Venous malformation following radiotherapy treatment	embolization
Hacein-Bey et al., 2013 (8)	Case Report	1 month; dysphagia, cough, vocal cord paralysis; trapezius muscle atrophy	Internal carotid artery (cervical portion); mass effect on cranial nerves X and XI	Pseudoaneurysm	Basilar skull fracture (Force for Down-fracture; PTJ separation)	Embolization; installation of a stent in the internal carotid artery.
Heggie et al., 2020 (29)	Retrospective study	1. 9 POD; Bone exposure in the maxillary vestibule 2. 1 POD; ischemia in the anterior maxillary gingiva 3. 1 POD; ischemia and bone exposure in the anterior maxillary vestibule 4. 1 POD; ischemia in the anterior maxillary gingiva 5. IPP; necrotic maxillary gingival tissue	Unclear or unspecified	Partial avascular necrosis in the maxilla	NR	1. Removal of the palatal device; subsequent reconstruction with bone graft 2. Hyperbaric oxygen therapy; subsequent placement of implants 3. Hyperbaric oxygen therapy 4. Hyperbaric oxygen therapy 5. Dental splinting; Hyperbaric oxygen therapy; subsequent rehabilitation with implants
Herdener et al., 2021 (26)	Case Report	4 POD; epistaxis; pallor, tachycardia, adynamia	Posterior lateral nasal and sphenopalatine arteries	Section/laceration	Nasal intubation	NP + Antifib Med+ Endoscopic cauterization
Humber et al., 2011 (36)	Case series	1. 8 POD; epistaxis and haemolacria 2. IPP; epistaxis and haemolacria	-	-	Surgical edema obstructing the Hasner valve	NP + proservation
Ito et al., 2023 (19)	Case Report	Tinnitus	Arteriovenous fistula that drains into the left pterygoid venous plexus from the left maxillary artery	Pseudoaneurysm	NR	A microcatheter was guided into the MMA, and the aneurysm and MMA were embolized with nine coils
Kashiya-ma and Hirano 2021 (15)	Case Report	8 POD; Nasal pain and epistaxis (recurring at 19 and 24 days)	Descending palatine artery	Pseudoaneurysm	NR	NP + embolization
Krishnan et al., 2011 (17)	Case Report	1 POD; weakness in extremities and sore throat; After 8 days: dysphagia, hoarseness, cough, difficulty breathing, tachycardia, lethargy	Internal carotid artery (ICA); Middle cerebral artery (MCA)	Pseudoaneurysm (ICA); ischemia (MCA)	NR	Tracheostomy + vascular stent + embolization
Kim et al., 2013 (16)	Case Report	14 POD; massive epistaxis; pallor, tremors, and mental confusion	Sphenopalatine artery	Pseudoaneurysm	NR	NP + Volume replacement (2PRBC + 3FFP) + embolization
Kufta et al., 2017 (35)	Case Report	IPP; Contralateral hemiparesis; hemifacial paralysis; dysarthria (stroke)	Internal carotid artery (middle cerebral artery - MCA)	Dissection of the internal carotid artery; MCA occlusion	Head movement during surgery	Decompressive craniectomy
Kumar et al., 2021 (9)	Case Report	9 POD; epistaxis (recurring at 1 and 7 days)	Distal branch of the maxillary artery e Sphenopalatine artery	Pseudoaneurysm	NR	NP + antifib med+ Volume replacement (2PRBC+ 4FFP) + embolization (in 2 instances)
Kumar et al., 2021 (18)	Case Report	45 POD; pain and swelling in the cheek; epistaxis (recurring at 14 and 21 days)	Superior posterior alveolar artery	Pseudoaneurysm	NR	NP + Volume replacement (2PRBC) + embolization
Kurasawa et al., 2022 (27)	Case Report	11 POD; severe epistaxis	Small vessels of the nasal mucosa	Incision/laceration	NR	NP + antifib med+ Endoscopic cauterization
Maleux et al., 2019 (10)	Case Report	7 POD; severe epistaxis (recurring within 1 day)	Sphenopalatine artery	Pseudoaneurysm	Inadequate separation of the nasal septum. Activation of the distractor device	embolization
Manafi et al., 2007 (11)	Case Report	10 POD; epistaxis (recurring at 22 and 12 days)	Internal maxillary artery	Pseudoaneurysm	NR	embolization
Niazi et al., 2018 (12)	Case Report	7 POD; oro-nasal bleeding	Descending palatine artery	Pseudoaneurysm	Moment of PTJ separation and use of a saw	NP + embolization
Osborne et al., 2017 (25)	Case Report	IPP; nausea, vomiting, lethargy, hemiplegia, hemiparesis, anisocoria, headaches	Internal carotid artery and internal jugular vein; middle cerebral artery (MCA)	Arteriovenous fistula; ischemia (MCA)	Moment of PTJ separation and force in Down-fracture	Decompressive craniectomy + fistula embolization
Park et al., 2019 (28)	Case Report	21 POD; severe epistaxis (recurring at 3 days)	Posterior lateral nasal artery and sphenopalatine artery	Section/laceration	NR	NP + Volume replacement in 2 instances (2PRBC/2PRBC) + Endoscopic cauterization
Pereira et al., 2010 (32)	Case Report	7 POD; maxillary mucosal ischemia	-	Partial avascular necrosis in the maxilla	Patient resumed smoking on the 3POD	Mouthwash with hydrogen peroxide + antibiotic therapy + hyperbaric oxygen therapy
Politis et al., 2012 (5)	Case series	1. 7 POD; epistaxis (recurring at 4, 4, and 14 days). 2. 7 POD; severe epistaxis 3. 1 POD; epistaxis	1. Branches of the maxillary artery 2 e 3. Not specified	None properly characterized	NR	1. NP; Volume replacement in 2 instances (4PRBC/2PRBC); embolization (in 2 instances) 2. Volume replacement (2PRBC) + Surgical exploration and hemostasis review 3. Surgical exploration and hemostasis review+ NP
Procopio et al., 2003 (4)	Case series	1. IPP; epistaxis (recurring at 21, 34, 63, 86, 108 days) 2. 5 POD; severe epistaxis (recurring at 7 days)	1. Descending palatine artery 2. Sphenopalatine artery	1 e 2. Pseudoaneurysm	1. Bony spur adjacent to the pterygoid process 2. Bony spur adjacent to the posterior wall of the maxillary sinus	1. NP; Volume replacement (2PRBC); embolization (in 2 instances); surgical exploration and hemostasis review + external carotid artery ligation 2. NP; Volume replacement (2PRBC); embolization
Smith et al., 2008 (24)	Case report	3 months; pulsatile tinnitus	Maxillary artery and pterygoid venous plexus	Arteriovenous fistula	Not specified	embolization
Singh et al., 2008(30)	Case report	IPP; gingival darkening and subsequent dental and alveolar bone loss	Not specified	Partial avascular necrosis in the maxilla	NR	Hyperbaric oxygen therapy
Teemul et al., 2017 (31)	Case report	7 POD; infected necrotic maxillary segment and oroantral fistula	Not specified	Partial avascular necrosis in the maxilla	Hypotensive anesthesia and sacrifice of one of the descending palatine arteries	Hyperbaric oxygen therapy + antibiotic therapy + pentoxifylline and tocopherol

Abbreviations: POD: postoperative day; IPP: immediate postoperative period; PRBC: packed red blood cells; FFP: fresh frozen plasma; MMA: middle meningeal artery; NR: not reported; NP: nasal packing; Antifib Med: antifibrinolytic medication; PTJ: pterygomaxillary junction.
